# Engineering Recombinant Reoviruses To Display gp41 Membrane-Proximal External-Region Epitopes from HIV-1

**DOI:** 10.1128/mSphere.00086-16

**Published:** 2016-05-18

**Authors:** Karl W. Boehme, Mine' Ikizler, Jason A. Iskarpatyoti, J. Denise Wetzel, Jordan Willis, James E. Crowe, Celia C. LaBranche, David C. Montefiori, Gregory J. Wilson, Terence S. Dermody

**Affiliations:** a Department of Pediatrics, Vanderbilt University Medical Center, Nashville, Tennessee, USA; b Elizabeth B. Lamb Center for Pediatric Research, Vanderbilt University Medical Center, Nashville, Tennessee, USA; c Department of Chemistry, Vanderbilt University, Nashville, Tennessee, USA; d Vanderbilt Vaccine Center, Vanderbilt University Medical Center, Nashville, Tennessee, USA; e Department of Pathology, Microbiology, and Immunology, Vanderbilt University Medical Center, Nashville, Tennessee, USA; f Department of Surgery, Duke University Medical Center, Durham, North Carolina, USA; g Department of Pathology, Duke University Medical Center, Durham, North Carolina, USA; h Laboratory for AIDS Vaccine Research and Development, Duke University Medical Center, Durham, North Carolina, USA; University of Pittsburgh School of Medicine

**Keywords:** human immunodeficiency virus, immunization, live vector vaccines, neutralizing antibodies, reovirus

## Abstract

The gp41 membrane-proximal external region (MPER) is a target for broadly neutralizing antibody responses against human immunodeficiency virus type 1 (HIV-1). However, replication-defective virus vaccines currently under evaluation in clinical trials do not efficiently elicit MPER-specific antibodies. Structural modeling suggests that the MPER forms an α-helical coiled coil that is required for function and immunogenicity. To maintain the native MPER conformation, we used reverse genetics to engineer replication-competent reovirus vectors that displayed MPER sequences in the α-helical coiled-coil tail domain of viral attachment protein σ1. Sequences in reovirus strain type 1 Lang (T1L) σ1 were exchanged with sequences encoding HIV-1 strain Ba-L MPER epitope 2F5 or the entire MPER. Individual 2F5 or MPER substitutions were introduced at virion-proximal or virion-distal sites in the σ1 tail. Recombinant reoviruses containing heterologous HIV-1 sequences were viable and produced progeny yields comparable to those with wild-type virus. HIV-1 sequences were retained following 10 serial passages in cell culture, indicating that the substitutions were genetically stable. Recombinant viruses engineered to display the 2F5 epitope or full-length MPER in σ1 were recognized by purified 2F5 antibody. Inoculation of mice with 2F5-containing vectors or rabbits with 2F5- or MPER-containing vectors elicited anti-reovirus antibodies, but HIV-1-specific antibodies were not detected. Together, these findings indicate that heterologous sequences that form α-helices can functionally replace native sequences in the α-helical tail domain of reovirus attachment protein σ1. However, although these vectors retain native antigenicity, they were not immunogenic, illustrating the difficulty of experimentally inducing immune responses to this essential region of HIV-1.

**IMPORTANCE** Vaccines to protect against HIV-1, the causative agent of AIDS, are not approved for use. Antibodies that neutralize genetically diverse strains of HIV-1 bind to discrete regions of the envelope glycoproteins, including the gp41 MPER. We engineered recombinant reoviruses that displayed MPER epitopes in attachment protein σ1 (REO-MPER vectors). The REO-MPER vectors replicated with wild-type efficiency, were genetically stable, and retained native antigenicity. However, we did not detect HIV-1-specific immune responses following inoculation of the REO-MPER vectors into small animals. This work provides proof of principle for engineering reovirus to express antigenic epitopes and illustrates the difficulty in eliciting MPER-specific immune responses.

## Abstract (precis)

Vaccines to protect against HIV-1, the causative agent of AIDS, are not approved for use. Antibodies that neutralize genetically diverse strains of HIV-1 bind to discrete regions of the envelope glycoproteins, including the gp41 MPER. We engineered recombinant reoviruses that displayed MPER epitopes in attachment protein σ1 (REO-MPER vectors). The REO-MPER vectors replicated with wild-type efficiency, were genetically stable, and retained native antigenicity. However, we did not detect HIV-1-specific immune responses following inoculation of the REO-MPER vectors into small animals. This work provides proof of principle for engineering reovirus to express antigenic epitopes and illustrates the difficulty in eliciting MPER-specific immune responses.

## INTRODUCTION

Despite tremendous strides in understanding human immunodeficiency virus type 1 (HIV-1) pathobiology since its discovery in 1983, an efficacious HIV-1 vaccine remains elusive. With an estimated 7,000 to 8,000 new infections occurring each day, an HIV-1 vaccine is our best hope for significantly reducing the global health burden attributable to HIV-1 (http://www.unaids.org). However, FDA-approved vaccines for HIV-1 are not available.

**FIG 1:**
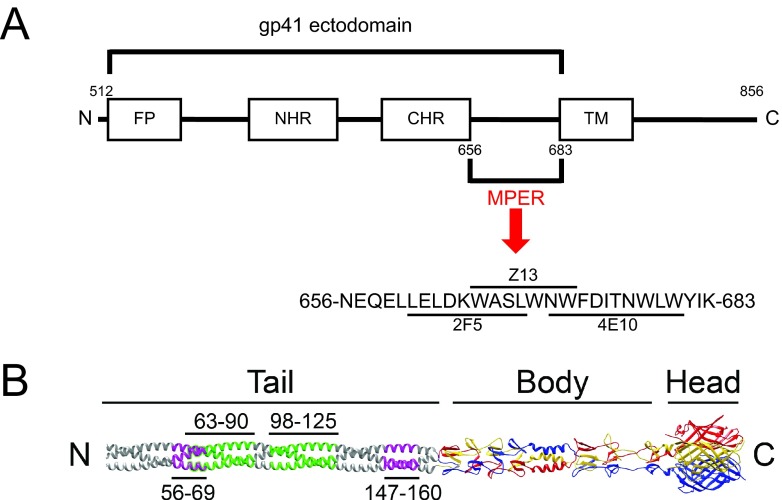
Structure of reovirus attachment protein σ1. (A) Schematic of the HIV-1 gp41 ectodomain. The gp41 ectodomain is comprised of a fusion peptide (FP), N-terminal heptad repeat (NHR), C-terminal heptad repeat (CHR), and membrane-proximal external region (MPER) (8). The transmembrane region also is indicated. MPER-specific 2F5, 4E10, and Z13 MAb epitopes are indicated. N- and C-terminal boundaries are shown. Numbers represent amino acid positions. (B) A full-length depiction of σ1, generated by appending a predicted trimeric α-helical coiled coil to the amino terminus of the largest available crystallized σ1 fragment (30). The three monomers of the crystallized region are shown in red, blue, and yellow; the model is shown in gray. Tail, body, and head regions are indicated. N and C termini are shown. Insertion sites for 2F5 sequences are indicated in purple. Insertion sites for whole MPER sequences are indicated in green.

Some HIV-1-infected persons produce monoclonal antibodies (MAbs) that neutralize diverse strains of HIV-1 (i.e., broadly neutralizing). These MAbs bind epitopes in the gp120 surface glycoprotein or gp41 transmembrane glycoprotein (1–6). Those that bind the gp41 membrane-proximal external region (MPER) can potently neutralize a wide spectrum of HIV-1 primary isolates, including strains in clades B and C (2, 7–9). Of three such MPER-specific MAbs, 2F5 and 4E10 bind to adjacent MPER epitopes and Z13 binds to an epitope overlapping the 2F5 and 4E10 binding sites (Fig. 1A) (8).

MPER sequences are conserved across HIV-1 subtypes (7, 8) and mediate an essential function in HIV-1 cell entry (8, 10). Following the sequential binding of gp120 to CD4 and a chemokine coreceptor, gp41 undergoes a series of conformational changes that result in fusion of the viral envelope and cell membrane (8). The ectodomain of gp41 is composed of a fusion peptide, an N-terminal heptad repeat (NHR), a C-terminal heptad repeat (CHR), and the MPER. Structural modeling suggests that the NHR, CHR, and MPER form α-helical coiled-coils that are required for function and immunogenicity (Fig. 1A) (8).

Three lines of evidence support a role for MPER-specific antibody responses in defense against HIV-1 infection. First, rates of mother-to-child transmission correlate inversely with reactivity of maternal sera against peptides corresponding to the MPER and C-terminal heptad repeat of gp41 (11–13). Second, MPER 2F5 epitope-specific IgA derived from colostral and cervico-vaginal secretions of HIV-infected women prevents transcytosis of HIV-1 across epithelial barriers *in vitro* (14). Third, passive immunization of nonhuman primates with MPER-specific MAbs isolated from HIV-1 subtype B-infected individuals, including 2F5 and 4E10, protects against infection or disease progression following simian-human immunodeficiency virus challenge (15–17). Despite these findings, a vaccine that induces protective MPER-specific antibody responses in experimental animals has not been developed.

Mammalian orthoreovirus (reovirus) forms nonenveloped icosahedral particles composed of two protein shells (18) that enclose 10 segments of double-stranded RNA (dsRNA) (19). The outer capsid contains four structural proteins, σ1, σ3, μ1, and λ2. The σ1 protein is anchored into pentameric λ2 turrets at the capsid vertices (18) and mediates reovirus attachment to target cells (20, 21). Virtually all mammals, including humans, can be infected with reovirus, but disease is restricted to the very young (22). Infection with reovirus is common, as the majority of adults have detectable reovirus-specific immune responses (23–25).

Reovirus attachment protein σ1 is a filamentous trimer that is ~480 Å in length (Fig. 1B) (26–28). It has a modular organization with three tandemly arranged structural regions: an N-terminal amphipathic α-helical coiled-coil tail (residues 1 to ~170), a triple β-spiral body interrupted by a short α-helix region (residues ~170 to 309), and a C-terminal globular head (residues 310 to 455) (28–30). Like other amphipathic α-helices, the α-helical coiled coil in the σ1 tail is formed by recurring sets of 7 amino acids, called heptad repeats (31). There are 25 heptad repeats in the α-helical coiled-coil region of strain type 1 Lang (T1L) σ1, spanning amino acid residues 7 to 181 (29). The MPER assumes an α-helical secondary structure (8) similar to that predicted for the σ1 tail (27–29).

Viable reovirus can be recovered from cells expressing T7 polymerase following transfection of plasmid cDNA copies of the viral gene segments under transcriptional control of the T7 promoter (32, 33). Neither helper virus nor coexpression of viral replication proteins is required. Plasmid-derived virus recapitulates properties of native virus in all cell culture and *in vivo* models of reovirus infection studied to date. We have used a reverse genetics system to introduce changes into viral capsid and replication proteins to define roles of individual amino acids, functional domains, and structural motifs in receptor utilization (30, 34–36), virion disassembly (32, 37, 38), membrane penetration (39, 40), interferon induction (41–43), dsRNA synthesis (44–46), viral replication and spread *in vivo* (32, 47–50), and neurovirulence (39, 40, 51). Thus, the technology exists to use reovirus as a replication-competent vaccine vector.

In this study, we recovered reovirus vectors in which α-helical regions in the σ1 tail were replaced with antigenic α-helical regions of the HIV-1 MPER (REO-MPER vectors). REO-MPER vectors replicated comparably to wild-type virus, were genetically stable following 10 cell culture passages, and retained the native MPER-specific MAb epitope. However, the REO-MPER vectors did not elicit HIV-1-specific immune responses in mice or rabbits. These findings indicate that reovirus can be engineered to serve as a vaccine vector, and they provide another example of the challenge in raising MPER-specific immune responses.

## RESULTS

### Generation of recombinant reoviruses displaying the 2F5 epitope in viral attachment protein σ1.

Reovirus attachment protein σ1 is partitioned into tail, body, and head domains (28–30) (Fig. 1B). To test the hypothesis that α-helical coiled-coil sequences derived from other viruses can be inserted into structurally homologous regions of the σ1 tail and retain native immunogenicity, we replaced two heptad repeats in σ1 with two heptad repeats from the HIV-1 gp41 MPER (Fig. 1B). The MPER sequences chosen for insertion contain the 2F5 epitope, which in the context of HIV-1 elicits broadly neutralizing antibody responses against HIV-1 (52–54). We replaced nucleotides encoding amino acids 56 to 69 or 147 to 160 of strain T1L σ1 with sequences encoding residues 656 to 669 of gp160 (the precursor of gp41) from HIV-1 strain Ba-L (656-NEQELLELDKWASL-669) in a σ1-encoding S1 gene plasmid vector. This region spans the entire 2F5 core epitope (662-ELDKWASL-669). Resultant plasmids were used to generate recombinant strain (rs) reoviruses via reverse genetics (32, 33). 2F5-expressing reoviruses rsT1L/σ1 2F5-56 and rsT1L/σ1 2F5-147 and wild-type rsT1L were isolated from cell lysates by plaque purification using murine L cells and purified using CsCl gradients. Insertion of the 2F5-encoding sequence into the viral genome was confirmed by sequence analysis of viral dsRNA.

**FIG 2:**
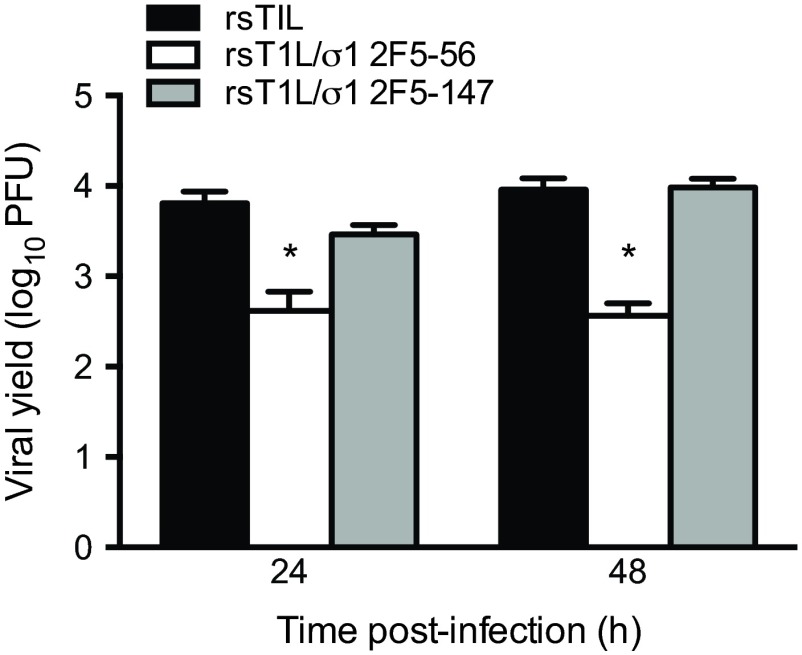
Reoviruses that display the HIV-1 2F5 epitope in attachment protein σ1 replicate efficiently in cell culture. L cells were adsorbed with rsT1L, rsT1L σ1/2F5-56, or rsT1L σ1/2F5-147 at an MOI of 1 PFU/cell. Viral titers in cell lysates were determined by plaque assay at 0, 24, and 48 h postadsorption. Results are expressed as the mean viral yield for triplicate samples. Error bars indicate standard deviations. *, *P* < 0.05 compared with rsT1L (Student’s *t* test).

To determine whether the substituted 2F5 sequences affect replication of the recombinant reoviruses, we quantified viral yields following infection of L cells (Fig. 2). Progeny yields of rsT1L/σ1 2F5-147 were comparable to those obtained for wild-type rsT1L at 24 and 48 h postinfection. However, yields of rsT1L/σ1 2F5-56 were markedly reduced relative to rsT1L. Sequence alterations were not detected in the S1 gene of either 2F5-containing virus following 10 serial passages in culture (data not shown). Together, these data indicate that α-helical sequences in σ1 can be replaced with heterologous sequences that contain a similar secondary structure and that the substitutions are genetically stable.

**FIG 3:**
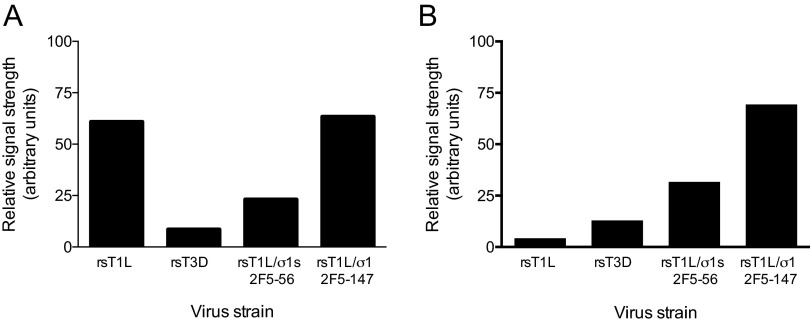
The 2F5 epitope is displayed by reovirus vectors encoding single 2F5 substitutions. Wells of FLISA plates were coated with 10^11^ particles of rsT1L, rsT3D, rsT1L σ1/2F5-56, or rsT1L σ1/2F5-147 and incubated with a T1L σ1 head-specific polyclonal antiserum (A) or MAb 2F5 (B) at 37°C for 1 h. Antibody binding to the immobilized virus was detected following incubation with fluorophore-conjugated donkey anti-human IgG. FLISA signals were quantified using a LI-COR Odyssey infrared imaging system.

To determine whether the 2F5 epitope is exposed on the surface of recombinant reovirus particles, we used a fluorophore-linked immunosorbent assay (FLISA) to detect the binding of MAb 2F5 to immobilized reovirus virions (Fig. 3). In these experiments, MAb 2F5 bound to viruses with insertions at either site in the σ1 α-helical coiled-coil domain but not to rsT1L. Both 2F5-expressing viruses also were detected by FLISA using a T1L σ1 head-specific antiserum (Fig. 3). These data indicate that the HIV-1 2F5 epitope retains the native conformation in the context of the reovirus σ1 protein.

### Immunogenicity of REO-2F5 vectors.

**FIG 4:**
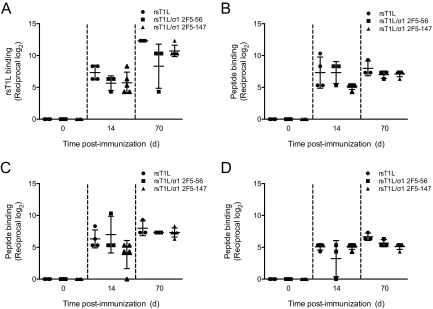
Induction of humoral immune responses in mice by REO-2F5 vectors. Six-week-old, reovirus-seronegative BALB/c mice were inoculated perorally with 10^7^ PFU of rsT1L, rsT1L/σ1 2F5-56, or rsT1L/σ1 2F5-147 (*n* = 3 to 5 mice per group). Blood was collected on day 0 (preinoculation) and on days 14 and 70 (postinoculation). Serial 4-fold dilutions of sera were tested for reovirus-specific antibodies using wells coated with rsT1L (A) or 2F5-specific antibodies using wells coated with three different 2F5-containing peptides (8926, 8927, or 8888) (B, C, and D) by FLISA. FLISA signals were quantified using a LI-COR Odyssey infrared imaging system and are expressed as the log_2_ mean reciprocal antibody titers relative to day 0 results. Error bars indicate standard deviations.

To determine whether the REO-2F5 vectors elicit 2F5-specific humoral immune responses, we inoculated BALB/c mice perorally with 10^7^ PFU of rsT1L, rsT1L/σ1 2F5-56, or rsT1L/σ1 2F5-147. Booster doses were administered 21 and 42 days following the initial immunization. Blood was collected on the day of inoculation (day 0) and on days 14 and 70 postinoculation. Pre- and postimmunization serum samples were tested for the presence of reovirus-specific and 2F5 peptide-specific antibodies by FLISA (Fig. 4). As anticipated, we detected significant increases in reovirus-specific antibody titers from day 14 to day 70 in sera from mice inoculated with wild-type virus and each REO-2F5 vector (Fig. 4A). Although sera from mice inoculated with rsT1L/σ1 2F5-56 or rsT1L/σ1 2F5-147 displayed detectable binding to two 2F5-containing peptides (Fig. 4B and C), the levels were comparable to those detected in sera from mice inoculated with rsT1L. Moreover, the 2F5 peptide binding activity in sera of mice inoculated with rsT1L, rsT1L/σ1 2F5-56, or rsT1L/σ1 2F5-147 did not exceed the levels of binding detected with the negative-control peptide (Fig. 4D).

**FIG 5:**
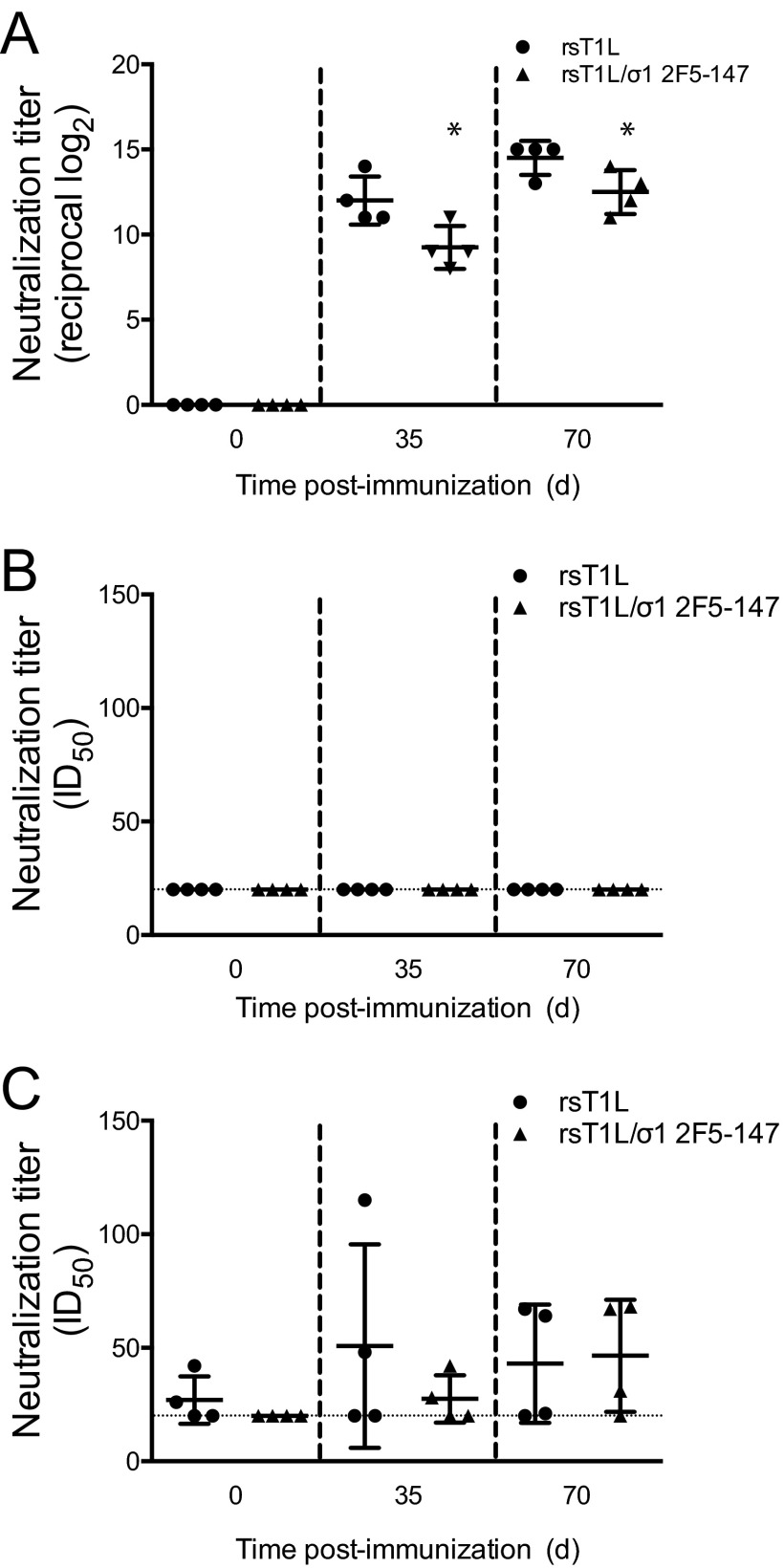
Induction of humoral immune responses in rabbits by REO-2F5 vectors. Six-week-old, reovirus-seronegative New Zealand White rabbits were inoculated perorally with 10^9^ PFU of rsT1L or rsT1L/σ1 2F5-147 (*n* = 4 rabbits per group). Blood was collected on day 0 (preinoculation) and on days 35 and 70 (postinoculation). Serial 4-fold dilutions of sera were tested for reovirus-specific antibodies by determining the PRNT60 (A) and HIV-specific antibodies by determining the capacity to neutralize infection of TZM-bl cells by tier 1 HIV-1 Env-pseudotyped viruses (B) or A3R5 cells by tier 2 HIV-1 Env-pseudotyped viruses (C). Results are expressed as the 50% infectious dose (ID_50_) for pseudotyped viruses. Error bars indicate standard deviations. *, *P* < 0.05 compared with rsT1L (Student’s *t* test).

We next tested whether the REO-2F5 vectors were capable of inducing 2F5-specific humoral immune responses in rabbits, which represent a preferred small-animal model for studies to evaluate induction of HIV-1-specific humoral immune responses (55). Rabbits were inoculated perorally with 10^9^ PFU of rsT1L or rsT1L/σ1 2F5-147. Booster doses were administered 21 and 42 days following the initial immunization. Blood was collected on the day of inoculation (day 0) and on days 35 and 56 postinoculation. Due to high levels of background binding of rabbit sera to 2F5 and control peptides, we tested pre- and postimmunization serum samples for the capacity to neutralize reovirus and HIV-1 (Fig. 5). Preimmunization samples were negative for reovirus-neutralizing antibodies. However, at day 35 postimmunization, all rabbits had detectable reovirus-specific antibodies, with 60% plaque reduction neutralization (PRNT60) antibody titers ranging from 8 to 14 (the log_2_ reciprocal titer) (Fig. 5A). Anti-reovirus neutralizing antibody titers increased slightly at day 70. A modest, but statistically significant, higher anti-reovirus antibody titer was detected in mice inoculated with rsT1L than in those inocluated with rsT1L/σ1 2F5-147. We used tier 1 and tier 2 Env-pseudotyped reporter retroviruses (56, 57) to assess the HIV-1 neutralization capacity in serum from reovirus-inoculated rabbits. No neutralization of tier 1 Env-containing pseudoviruses was detected in any of the samples tested. Neutralization activity against tier 2 Env-containing pseudotyped viruses was comparable between sera collected from rabbits inoculated with rsT1L or rsT1L/σ1 2F5-147 (Fig. 5B). Together, these findings indicate that REO-2F5 vectors elicit vector-specific antibodies but fail to stimulate production of HIV-1 2F5 epitope-specific humoral immune responses.

### Second-generation reovirus vectors encode the entire MPER.

We thought it possible that the minimal 2F5 epitope inserted into σ1 was of insufficient size to elicit a detectable response. To assess this possibility, we engineered second-generation reovirus vectors in which a sequence containing four heptad repeats corresponding to the entire MPER motif from HIV-1 strain Ba-L (656-NEQELLELDKWASLWNWFDITKWLWYIK-683) was substituted for four heptad repeats in the T1L σ1 protein. This region contains epitopes for three MPER-specific antibodies, 2F5 (662-ELDKWASL-669), 4E10 (669-LWNWFDITKWLWYIK-683), and Z13 (666-WASLWNWFDITK-677) (Fig. 1A). MPER-specific sequences were introduced into σ1 at residues 63 to 90, 98 to 125, or 147 to 174, and recombinant viruses (REO-MPER vectors) were recovered using reverse genetics (Fig. 1B). Viruses with MPER sequences inserted at residues 63 (rsT1L/σ1 MPER-63) and 98 (rsT1L/σ1 MPER-98) were viable. However, we were not able to recover virus containing the insertion at amino acid 147 (rsT1L/σ1 MPER-147). Insertion of the MPER-encoding sequence into the S1 gene segment of viable virus was confirmed by sequence analysis of viral dsRNA.

**FIG 6:**
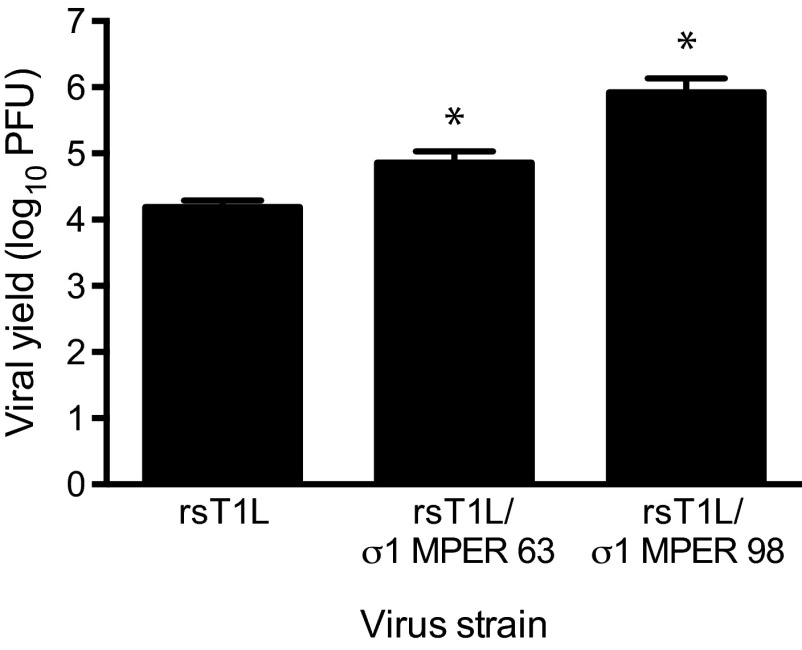
Reoviruses that display the HIV-1 MPER in attachment protein σ1 replicate efficiently in cell culture. L cells were adsorbed with rsT1L, rsT1L σ1/MPER-63, or rsT1L σ1/MPER-98 at an MOI of 1 PFU/cell. Viral titers in cell lysates were determined by plaque assay at 0 and 48 h postadsorption. Results are expressed as the mean viral yield for triplicate samples. Error bars indicate standard deviations. *, *P* < 0.05 compared with rsT1L (Student’s *t* test).

To determine whether insertion of the entire MPER sequence into the σ1 protein affects viral replication in cultured cells, we quantified viral titers 48 h after infection of L cells (Fig. 6). Both rsT1L/σ1 MPER-63 and rsT1L/σ1 MPER-98 produced yields equivalent to, or greater than, those of wild-type rsT1L. No sequence alterations were detected in the recombinant reovirus S1 gene sequences following 10 serial passages in cell culture. Together, these data indicate that the entire MPER of HIV-1 gp41 can be substituted for α-helical regions of σ1. However, the C-terminal portion of the σ1 tail is less amenable to genetic manipulation, suggesting that key determinants of σ1 protein stability or function reside in this region of the protein.

**FIG 7:**
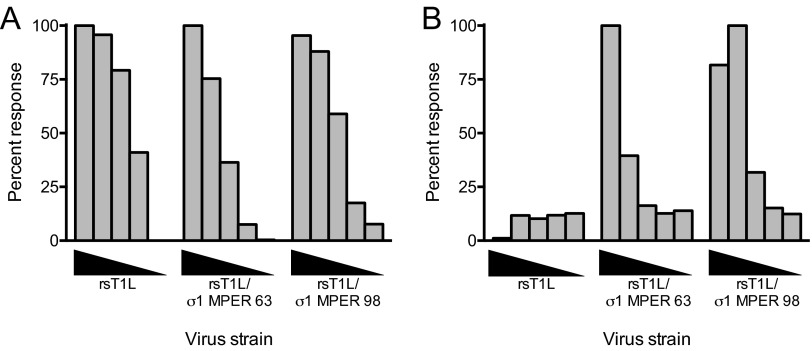
Second-generation reovirus vectors display the MPER epitope. L cells plated in 96-well plates were adsorbed with 10-fold serial dilutions of rsT1L, rsT1L/σ1 MPER-63, or rsT1L/σ1 MPER-98 over a range of MOIs (0.01 to 100 PFU/cell). At 24 h postadsorption, cells were fixed and stained with 2F5 epitope-specific monoclonal antibody (A) or polyclonal reovirus-specific antiserum (B) followed by secondary antibodies conjugated to AlexaFluor 488. The results were quantified using an Odyssey infrared imaging system and normalized to the maximum response achieved for each antibody. The data are presented as percentages of the normalized response.

To determine whether the MPER epitope in the REO-MPER vectors retains its native conformation, we stained L cells infected with rsT1L, rsT1L/σ1 MPER-63, or rsT1L/σ1 MPER-98 with a 2F5 epitope-specific monoclonal antibody (Fig. 7A). Cells infected with rsT1L/σ1 MPER-63 or rsT1L/σ1 MPER-98 were stained with the 2F5 antibody, but no 2F5 staining was observed in cells infected with rsT1L. As a positive control, cells infected with each virus displayed staining with polyclonal reovirus-specific antiserum (Fig. 7B). These data indicate that the HIV-1 MPER epitope retains its native conformation in the context of the reovirus σ1 protein.

### Immunogenicity of REO-MPER vectors displaying the entire MPER motif.

**FIG 8:**
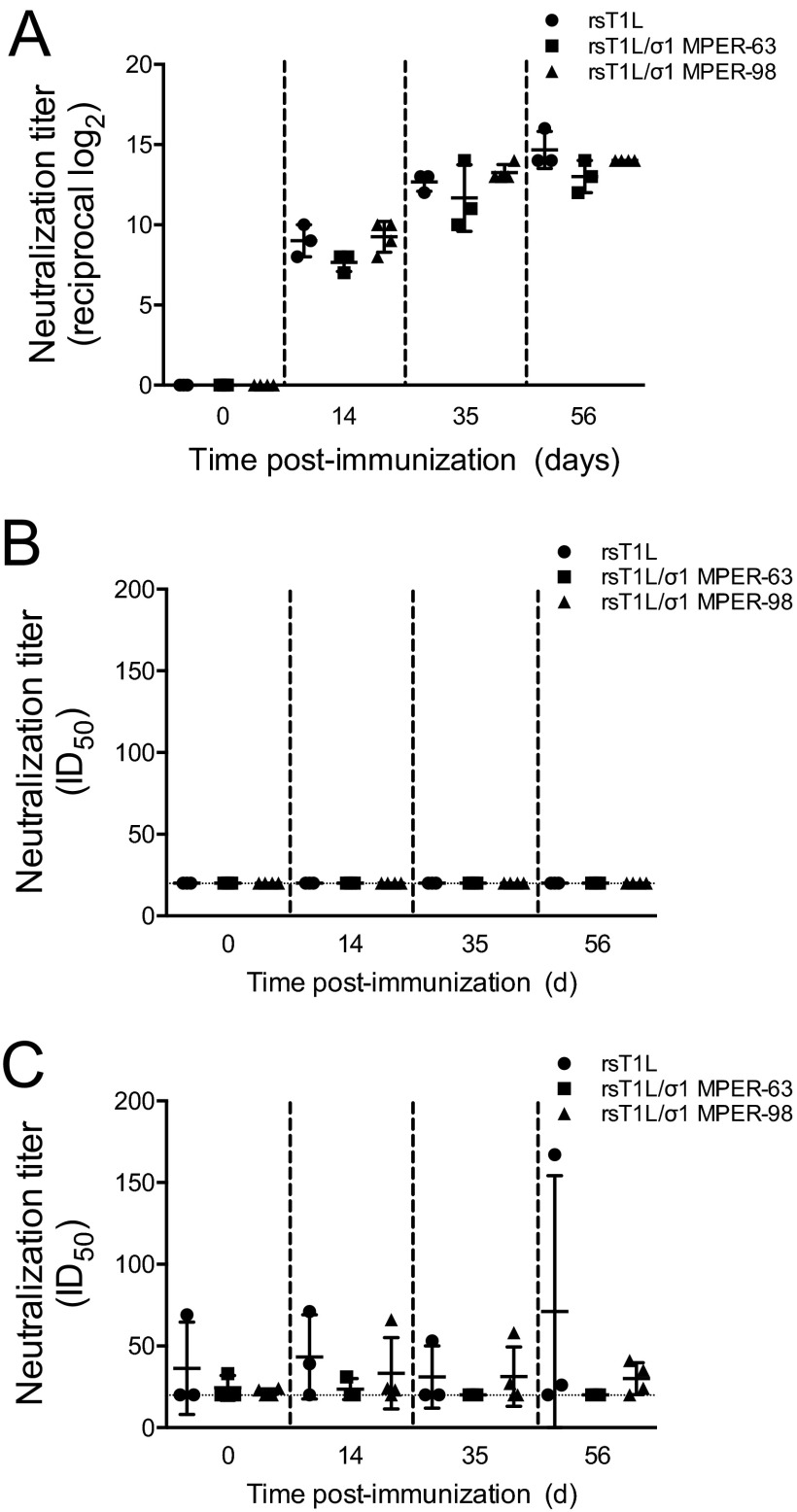
Immunogenicity of REO-MPER vectors. Six-week-old, reovirus-seronegative New Zealand White rabbits were inoculated perorally with 10^9^ PFU of T1L, rsT1L/σ1 MPER-63, or rsT1L/σ1 MPER-98 (*n* = 3 or 4 rabbits per group). Blood was collected on day 0 (preinoculation) and on days 14, 35, and 56 (postinoculation). Serial 4-fold dilutions of sera were tested for reovirus-specific antibodies by determining the PRNT60 (A) and HIV-specific antibodies by determining the capacity to neutralize infection of TZM-bl cells by tier 1 HIV-1 Env-pseudotyped viruses (B) or A3R5 cells by tier 2 HIV-1 Env*-*pseudotyped viruses (C). Results are expressed as the 50% infectious dose (ID_50_) for pseudotyped viruses. Error bars indicate standard deviations.

To determine whether REO-MPER vectors elicit MPER-specific humoral immune responses, we inoculated rabbits perorally with 10^9^ PFU of rsT1L, rsT1L/σ1 MPER-63, or rsT1L/σ1 MPER-98. Booster doses were administered 21 and 42 days following the initial immunization. Blood was collected on the day of inoculation (day 0) and on days 14, 35, and 56 postinoculation. Pre- and postimmunization serum samples were tested for the presence of reovirus-specific and MPER peptide-specific antibodies by FLISA (Fig. 8). As with the previous experiments, preimmunization samples were negative for both types of antibodies. At day 14 postimmunization, all rabbits had detectable reovirus-specific antibodies, with antibody titers ranging from 7 to 10 (reciprocal log_2_ titer) (Fig. 8A). By day 56, anti-reovirus antibody titers increased to a range of 12 to 16 (reciprocal log_2_ titer) per animal. However, we did not detect neutralization activity in serum from rabbits inoculated with MPER-containing vectors when we used tier 1 or tier 2 Env-pseudotyped viruses (Fig. 8B and C). Together, these findings indicate that REO-MPER vectors elicit reovirus-specific antibody responses but not responses specific for the HIV-1 MPER.

## DISCUSSION

Most current HIV-1 vaccine candidates are replication defective, administered intramuscularly, and unlikely to induce immune responses at mucosal surfaces. The goal of this study was to develop an orally administered replication-competent reovirus-based HIV-1 vaccine that stimulates mucosal and systemic humoral immune responses against broadly neutralizing epitopes of HIV-1. Reovirus infects intestinal mucosa to stimulate potent immune responses (58, 59), is naturally attenuated in humans (25, 60, 61), and can be manipulated to express vaccine antigens (32, 33). These features make reovirus an ideal HIV-1 vaccine vector.

In mice, reovirus initiates infection by traversing M cells overlying Peyer’s patches (PPs) in the small intestine (62, 63) and undergoes primary replication in adjacent epithelial cells and PP mononuclear cells (64–66). The reovirus-specific humoral immune response is characterized by mucosal IgA production through priming of B lymphocytes and development of plasma cells in PPs, mesenteric lymph nodes, and the spleen (67). IgA and IgG responses are directed against reovirus outer capsid proteins σ1, σ3, and μ1 (67–70), and reovirus-specific IgA and IgG antibodies prevent viral intestinal infection and systemic dissemination (68), respectively. Disease associated with reovirus infection is rare and limited to the very young, who recover without sequelae (22, 71–73). Preexisting immunity to reovirus is not a barrier to induction of immune responses, as demonstrated during clinical trials in which reovirus was used as an oncolytic adjunct to cancer chemotherapy (24).

We engineered reovirus vectors to display epitopes derived from the HIV-1 gp41 MPER that elicit broadly neutralizing antibodies. The resulting REO-MPER vectors replicated comparably to wild-type virus in cell culture, retained the inserted sequences following 10 serial cell culture passages, and were recognized by an MPER-specific MAb that neutralizes HIV-1 infection in cultured cells and protects against HIV-1 challenge in animal models. REO-MPER vectors elicit high-titer reovirus-specific antibody responses in mice and rabbits but fail to induce MPER-specific antibody responses or HIV-1 neutralization activity. Collectively, these data indicate that reovirus can be manipulated to display antigenic epitopes. However, the REO-MPER vectors used in this study did not yield detectable HIV-1-specific immune responses.

Several vector systems have been designed to display HIV-1 MPER sequences. These include adenovirus (74), hepatitis A virus (75), hepatitis B virus (76), influenza A virus (77), papillomavirus (78), potato virus X (79), and rhinovirus (80). However, these MPER-expressing vectors failed to elicit neutralizing antibody responses in animal models. In this regard, the MPER epitopes in each vector were displayed as monomeric units within surface-exposed loops of vector capsid components, whereas these epitopes form α-helical coiled-coil trimers in native gp41 (8). The strategy used here yielded vectors that displayed MPER sequences in the native trimeric conformation. Nonetheless, these vectors did not elicit MPER-specific immune responses.

What accounts for the failure of REO-MPER vectors to stimulate production of MPER-specific antibodies in mice and rabbits? It is possible that the epitope inserted into σ1 is not fully native. Although the σ1 tail domain may be sufficient to allow MPER epitopes to form trimers, additional structural constraints provided by the native gp41 framework could be required for the epitopes to be displayed in an immunogenic manner. We think this possibility is less likely, as MAb 2F5 is capable of binding REO-MPER vectors that display amino acid sequences that constitute the 2F5 epitope. It also is possible that generation of MPER-specific antibodies requires association with lipid. The MPER is immediately adjacent to the viral membrane (81), and some antibodies that recognize MPER epitopes, including 2F5 and 4E10, require interaction with lipid along with engagement of gp41 (82, 83). To our knowledge, σ1 does not associate with membranes and, therefore, it may be difficult to engineer MPER-containing σ1 molecules with the capacity to elicit MPER-specific antibodies if lipid engagement is required for immunogenicity. Alternatively, mice and rabbits may be incapable of producing an MPER-specific antibody response, even following three immunizations with a vector that appropriately displays the target epitope. MPER-specific antibodies in humans are characterized by substantial somatic hypermutation (84), which likely takes a prolonged interval (on the order of months to years) of chronic infection to develop (85, 86). The immunization protocols used in our study would not likely recapitulate the exposure of the MPER epitope required to generate an effective antibody response. Moreover, the CDR3 region of MPER-specific MAbs is much longer than the corresponding region of mouse or rabbit antibodies, which may preclude development of MPER-specific antibody responses in these animals (52, 87).

Our findings indicate that reovirus can be engineered to display foreign epitopes. The α-helical coiled-coil σ1 tail domain appears to tolerate insertion of heterologous sequences as long as the heptad repeat register is maintained. A common theme among enveloped viruses is utilization of viral proteins that contain α-helical motifs to mediate fusion of the viral envelope with the cell membrane during virus entry (31). For many human-pathogenic viruses, including Ebola virus (88) and influenza virus (89), broadly neutralizing antibodies have been isolated that bind to these α-helical coiled-coil regions and prevent infection. It is possible that reovirus vectors constructed to display these sequences will elicit protective immune responses.

## MATERIALS AND METHODS

### Cell lines.

L929 (L) cells were maintained in Joklik’s minimal essential medium (Lonza) supplemented to contain 5% fetal bovine serum (FBS), 2 mM l-glutamine, 100 U/ml penicillin, 100 µg/ml streptomycin, and 25 ng/ml amphotericin B. HeLa cells were maintained in Dulbecco’s modified Eagle medium (DMEM; Gibco) supplemented to contain 10% FBS, 2 mM l-glutamine, 100 U/ml penicillin, 100 µg/ml streptomycin, and 25 ng/ml amphotericin B (Invitrogen). BHK-T7 cells were maintained in DMEM supplemented to contain 5% FBS, 2 mM l-glutamine, 2% minimal essential medium amino acid solution (Invitrogen), and 1 mg/ml Geneticin (Invitrogen).

### Viruses.

Recombinant reoviruses were recovered by using plasmid-based reverse genetics (32, 33). Monolayers of BHK-T7 cells at approximately 90% confluence (3 × 10^6^ cells) in 60-mm dishes (Corning) were cotransfected with nine plasmid constructs representing cloned gene segments from strain T1L: pT7-L1T1L (2 µg), pT7-L2T1L (2 µg), pT7-L3T1L (2 µg), pT7-M1T1L (1.75 µg), pT7-M2T1L (1.75 µg), pT7-M3T1L (1.75 µg), pT7-S2T1L (1.5 µg), pT7-S3T1L (1.5 µg), and pT7-S4T1L (1.5 µg), in combination with 2 µg of pBacT7-S1T1L or S1-MPER constructs. For each transfection mixture, 3 µl of TransIT-LT1 transfection reagent (Mirus) was used per microgram of plasmid DNA. Following 2 days of incubation, recombinant virus was isolated from transfected cells by plaque purification using monolayers of L cells (90). To engineer recombinant viruses displaying MPER sequences in σ1, pBacT7-S1T1L was altered via QuikChange (Stratagene) site-directed mutagenesis. Nucleotides encoding amino acids 56 to 69 and 147 to 160 of T1L σ1 were exchanged with sequences encoding residues 656 to 669 of gp160 (the precursor of gp41) from HIV-1 strain Ba-L (656-NEQELLELDKWASL-669) spanning the entire 2F5 epitope. Nucleotides encoding amino acids 63 to 90, 98 to 125, and 147 to 174 (four heptad repeats) of T1L σ1 were exchanged with sequences encoding the entire MPER motif from HIV-1 strain Ba-L (656-NEQELLELDKWASLWNWFDITKWLWYIK-683). Sequences encoding whole MPER insertions of four heptad repeats were synthesized by GenScript, introduced into the PstI site of pUC57, and subcloned into pBacT7-S1 by using a TaKaRa DNA ligation kit (Clontech). Sequences of mutant viruses were confirmed using S1 gene cDNAs prepared from viral RNA extracted from purified virions subjected to OneStep reverse transcription-PCR (RT-PCR; Qiagen) with S1-specific primers. Primer sequences are available from the corresponding authors upon request. PCR products were analyzed following electrophoresis in Tris-borate-EDTA agarose gels or purified and subjected directly to sequence analysis.

Purified reovirus virions were prepared using second- or third-passage L cell lysate stocks of twice-plaque-purified reovirus, as described elsewhere (26). Viral particles were freon extracted from infected cell lysates, layered onto 1.2- to 1.4-g/cm^3^ CsCl gradients, and centrifuged at 62,000 × *g* for 18 h. Bands corresponding to virions (1.36 g/cm^3^) (91) were collected and dialyzed in virion storage buffer (150 mM NaCl, 15 mM MgCl_2_, 10 mM Tris-HCl [pH 7.4]). The concentration of reovirus virions in purified preparations was determined from the equivalence of one optical density (OD) unit at 260 nm to 2.1 × 10^12^ virions (91). Viral titers were determined by plaque assay using L cells (90).

### Virus replication assays.

For assays of viral yield, L cells (5 × 10^4^ cells/well) seeded in 24-well plates (Costar) were adsorbed in triplicate with reovirus strains at a multiplicity of infection (MOI) of 1 PFU/cell at room temperature for 1 h in serum-free medium, washed once with Dulbecco’s phosphate-buffered saline without calcium or magnesium (PBS; Invitrogen), and incubated in serum-containing medium for various intervals. Cells were frozen and thawed twice prior to determination of viral titer by plaque assay using L cells (90). Viral yield was calculated using the following formula: log_10_ yield at *t*_x_ = log_10_(PFU/ml)*t*_x_ − log10(PFU/ml)*t*_0_, where *t* is the time postadsorption.

### Serial passage of reovirus vectors in cell culture.

Recombinant viruses were consecutively passaged 8 to 10 times in confluent monolayers of L cells cultivated in T-25 tissue culture flasks (Costar) by inoculating cells with 1 ml of virus stock diluted to provide a low MOI. Cells were lysed by freezing and thawing twice, and viral RNA from each passage was purified from culture lysates by using an RNA isolation kit (Roche). RNA was converted to cDNA via OneStep RT-PCR (Qiagen) using S1-specific primers, and cDNAs were sequenced.

### Detection of MPER epitopes by FLISA.

Black, clear-bottom, 96-well plates (Costar) were coated with 10^10^ or 10^11^ particles of either rsT1L or recombinant reovirus strains and incubated with 2.5 µg/ml of MAb 2F5 (92) and rabbit σ1 head-specific antiserum (47) at 37°C for 1 h. Antibody binding to the 2F5 epitope displayed by immobilized virus was detected following incubation with fluorophore-conjugated donkey anti-human IgG (Rockland). Antibody binding to the σ1 head was detected following incubation with goat anti-rabbit antiserum (LI-COR). FLISA signals were quantified using a LI-COR Odyssey infrared imaging system and are expressed as the relative signal strength, proportional to the quantity of dye-labeled antibody bound per well. Background fluorophore-conjugated antibody binding to empty wells was subtracted from that in particle-coated wells to determine the final integrated intensity values.

### Infection of mice and rabbits.

Reovirus-seronegative BALB/c mice were obtained from Jackson Laboratory. Fifteen 6-week-old mice were inoculated with wild-type rsT1L, rsT1L/σ1 2F5-56, or rsT1L/σ1 2F5-147 diluted in PBS on day 0 and were boosted on days 21 and 42. Blood samples were obtained by tail vein puncture on day 0 (prior to inoculation) and on days 14 and 70. The dose of reovirus used for all mouse inoculations ranged from 10^6^ to 10^7^ PFU/ml.

Reovirus-seronegative New Zealand White rabbits were obtained from Covance, Inc. Purified recombinant reovirus was emulsified in TiterMax gold adjuvant (Sigma-Aldrich) for the first experiment and PBS for the subsequent experiment. For the first experiment, eight 14-week-old rabbits were inoculated intramuscularly in the hind limb with rsT1L or rsT1L/σ1 2F5-147 on day 0 and boosted on days 21 and 42. Blood samples were obtained by ear central artery puncture on days 0 (prior to immunization), 14, 35, 56, and 70. For the second experiment, 10 14-week-old rabbits were inoculated intramuscularly in the hind limb with rsT1L, rsT1L/σ1 MPER-63, or rsT1L/σ1 MPER-98 on day 0 and boosted on days 21 and 42. Blood samples were collected on days 0 (prior to immunization), 14, 35, and 56. The dose of reovirus used for all rabbit inoculations ranged from 10^8^ to 10^9^ PFU/ml.

Animal husbandry and experimental procedures were performed in accordance with Public Health Service policy and approved by the Vanderbilt University School of Medicine Institutional Animal Care and Use Committee.

### Quantification of reovirus-specific antibody responses.

Reovirus-specific antibody responses were determined in PRNT60 assays with rsT1L and L cells. Serum samples were heat inactivated at 56°C for 30 min, serially diluted 4-fold beginning with a dilution of 1:20, and incubated with an equal volume of a virus stock containing 100 PFU. After incubation for 1 h, the serum-virus mixtures were inoculated in duplicate onto confluent L-cell monolayers in 6-well tissue culture plates (Costar). Cells were stained with neutral red on day 7, and plaques were enumerated (90). Serum reciprocal geometric mean titers capable of reducing plaque counts by 60% were calculated via regression analysis. Seroconversion was defined as a ≥4-fold increase in serum neutralizing antibody titer to rsT1L at study days 14, 35, 56, and 70 compared with the prevaccination PRNT60.

### Detection of 2F5 epitope-specific antibodies by immunofluorescence.

Pre- and postimmunization serum samples from vaccine recipient and control animals were evaluated for MPER-specific antibodies by FLISA and kinetic enzyme-linked immunosorbent assay (ELISA) (93) using a reference panel of HIV-1 MPER-specific peptides (15-mers; NIH AIDS Research and Reference Reagent Program). Duplicate wells were coated with 2F5 epitope-containing peptides 8926 (EQELLELDKWASLWN), 8927 (LELDKWASLWNWFDI), or negative-control peptide 8888 (VVQREKRAVGIGAMF). An uncoated well was included with each test sample to determine the background level of binding, which was subtracted from the test sample result. A standard curve prepared using a positive rabbit antiserum (provided by G. Ofek, NIH) was included in each assay as a positive control. The kinetic ELISA was developed by addition of biotinylated goat anti-rabbit IgG conjugated with streptavidin-horseradish peroxidase, followed by addition of the substrate 2,2′-azino-bis(3-ethylbenzothiazoline-6-sulfonic acid) diammonium salt (ABTS). Binding was quantified using a Molecular Devices microplate reader and Molecular Devices Softmax software.

### Quantification of HIV-1-neutralizing antibody responses.

Serum samples from rabbits were evaluated for the capacity to neutralize a tier 1 HIV-1-Env*-*containing pseudovirus (W61D TCLA.71) in a TZM-bl assay (56) or a tier 2 HIV-1-Env*-*containing *Renilla* luciferase-encoding infectious molecular clone (WITO.LucR.T2A.ecto) using A3R5 cells (57, 94). Reagents were obtained from the NIH AIDS Research and Reference Reagent Program. These assays were performed in a biosafety level 3 laboratory.

### Statistical analysis.

Means of triplicate samples were compared by using an unpaired Student’s *t* test or one-way analysis of variance (ANOVA). Statistical analyses were performed using Prism 6.0 software (GraphPad Software, Inc.). *P* values of <0.05 were considered statistically significant.

### Preparation of the σ1 schematic.

The full-length schematic of σ1 (Fig. 1B) was prepared using the Chimera program from the University of California, San Francisco (http://www.rbvi.ucsf.edu/chimera).
